# Urinary metabolomics analysis based on LC-MS for the diagnosis and monitoring of acute coronary syndrome

**DOI:** 10.3389/fmolb.2025.1547476

**Published:** 2025-04-09

**Authors:** Jiaqi Liu, Aiwei Wang, Feng Qi, Xiaoyan Liu, Zhengguang Guo, Haidan Sun, Mindi Zhao, Tingmiao Li, Fei Xue, Hai Wang, Wei Sun, Chengyan He

**Affiliations:** ^1^ Department of Laboratory Medicine, China-Japan Union Hospital of Jilin University, Changchun, China; ^2^ Institute of Basic Medical Sciences, Chinese Academy of Medical Sciences, School of Basic Medicine, Peking Union Medical College, Beijing, China; ^3^ Department of Laboratory Medicine, Beijing Hospital, National Center of Gerontology, Institute of Geriatric Medicine, Chinese Academy of Medical Sciences, Beijing, China

**Keywords:** acute coronary syndrome, biomarker, metabolomics, urine, non-targeted

## Abstract

**Background:**

Acute coronary syndrome (ACS) is a cardiovascular disease caused by acute myocardial ischemia. The aim of this study was to use urine metabolomics to explore potential biomarkers for the diagnosis of ACS and the changes in metabolites during the development of this disease.

**Methods:**

Urine samples were collected from 81 healthy controls and 130 ACS patients (103 UA and 27 AMI). Metabolomics based on liquid chromatography-mass spectrometry (LC-MS) was used to analyze urine samples. Statistical analysis and functional annotation were applied to identify potential metabolite panels and altered metabolic pathways between ACS patients and healthy controls, unstable angina (UA), and acute myocardial infarction (AMI) patients.

**Results:**

There were significant differences in metabolic profiles among the UA, AMI and control groups. A total of 512 differential metabolites were identified in this study. Functional annotation revealed that changes in arginine biosynthesis, cysteine and methionine metabolism, galactose metabolism, sulfur metabolism and steroid hormone biosynthesis pathways occur in ACS. In addition, a panel composed of guanidineacetic acid, S-adenosylmethionine, oxindole was able to distinguish ACS patients from healthy controls. The AUC values were 0.8339 (UA VS HCs) and 0.8617 (AMI VS HCs). Moreover, DL-homocystine has the ability to distinguish between UA and AMI, and the area under the ROC curve is 0.8789. The metabolites whose levels increased with disease severity the disease were involved mainly in cysteine and methionine metabolism and the galactose metabolism pathway. Metabolites that decrease with disease severity are related mainly to tryptophan metabolism.

**Conclusion:**

The results of this study suggest that urinary metabolomics studies can reveal differences between ACS patients and healthy controls, which may help in understanding its mechanisms and the discovery of related biomarkers.

## 1 Introduction

Acute coronary syndrome (ACS) is a complex clinical syndrome caused by acute myocardial ischemia and is one of the leading causes of death worldwide (Surendran et al., 2021). The main subtypes of ACS include unstable angina (UA), non-ST-segment elevation myocardial infarction (NSTEMI) and ST-segment elevation myocardial infarction (STEMI) (Reed et al., 2017). Rupture or erosion of unstable atherosclerotic plaques, inflammatory responses, and acute intracoronary thrombosis are thought to be the primary pathologic basis for most patients with ACS (Bergmark et al., 2022; Crea and Libby, 2017; Davies and Thomas, 1984; Wolf and Ley, 2019; Crea and Liuzzo, 2013). Currently, the various subtypes of ACS need to be differentiated by combining clinical presentation, ECG and markers of myocardial injury (Bhatt et al., 2022). However, biomarkers such as troponin may be elevated in other diseases (Karády et al., 2021), and the identification of different subtypes requires continuous testing of ECG and myocardial injury markers (Westermann et al., 2017).

With the development of high-throughput metabolomics techniques, plasma metabolomics has been used to study acute coronary syndromes. Plasma metabolites have been used asbiomarkers for a variety of diseases, including cancer, autoimmune diseases, and cardiovascular disease (Vallejo et al., 2009; Liu et al., 2023). In 2014 Laborde et al. (2014) applied gas chromatography-mass spectrometry (GC-MC) techniques to analyze peripheral plasma from patients with non-ST-segment elevation ACS and healthy controls and identified 15 metabolites. Potential biomarkers consisting of 5-OH-tryptophan, 2-OH-butyric acid, and 3-OH-butyric acid were identified in the validation group as indicators of oxidative stress and hypoxia in cardiomyocytes. Late Fan et al. (2016) collected plasma samples from 2,324 patients with coronary heart disease (CHD) from 4 independent centers for metabolomic analysis, and changes in metabolic pathways, such as phospholipid catabolism and the tricarboxylic acid cycle, were detected in the disease group.

Urine is a proximal biological fluid, and urine-based proteomics and metabolomics have also been widely used in biomarker discovery and clinical applications (Thomas et al., 2016). As early as 2014, Martin-Lorenzo et al. (2015) reported that the urine metabolites 1-methylhydantoin, and 2-hydroxyphenilacetic acid were downregulated in patients with ACS upon admission and returned to normal upon discharge, these findings could be used as potential biomarkers for the diagnosis of ACS. In 2018 Wang et al. (2018) applied ultra-performance liquid chromatography/mass spectrometry (UPLC/MS) technique to the urine of ACS patients and healthy controls, and 20 biomarkers and 9 metabolic pathways were obtained.

Previous studies have shown that it is feasible to understand changes in diseased organisms through metabolomic analysis of body fluids. However, most of the related articles have focused on the blood metabolome of patients with acute coronary syndrome, and fewer studies have focused on urine to further explore the physiopathological changes in acute coronary syndrome. There are several advantages to using urine for metabolomics analysis. First, urine is noninvasive, does not require venipuncture, avoids pain, infection, or bleeding risk, and is especially suitable for patients receiving anticoagulant therapy and coagulopathy. Second, it increases the feasibility of continuous monitoring, allowing frequent sampling to facilitate dynamic observation of disease changes and real-time tracking of metabolic changes after myocardial ischemia or reperfusion (Sarafidis et al., 2017). Finally, the cost of urine collection tubes and storage equipment is usually low (Borgatta et al., 2021), and urine does not require anticoagulation, centrifugation or complex processing, which can be completed by the patient, which is suitable for remote, resource-poor areas or delayed detection.

**FIGURE 1:**
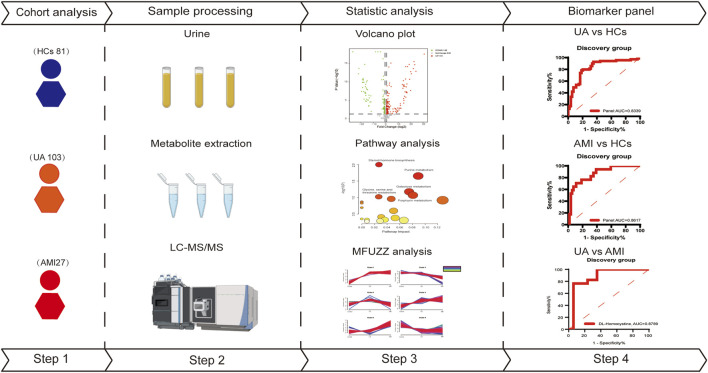
The workflow of this study.

In this study, we used LC-MS to analyze urine samples from 130 patients with acute coronary syndrome (103 UA and 27 AMI) and 81 healthy patients. Patients with acute myocardial infarction (AMI) can be systematically subdivided into ST-segment elevation myocardial infarction and non-ST-segment elevation myocardial infarction (Reed et al., 2017; Deckers, 2013). Therefore, the AMI patients in this study consisted of 19 NSTEMI patients and 8 STEMI patients. First urine samples from 69 UA patients and 17 AMI patients and 54 healthy patients were analyzed to identify differences between different subtypes of ACS patients and healthy patients, which were used to define potential metabolic biomarkers to diagnose ACS. AMI is associated with more myocardial damage than is UA, and the risk of short-term death is significantly greater than that of UA (Anonymous, 2000). Moreover, UA that is not treated in a timely manner may progress to AMI (Fu et al., 2023). All of these findings indicate the urgent need to distinguish UA from AMI early to select different treatment strategies. In order to better distinguish between UA and AMI, we also analyzed the changes in urine metabolites in age and sex matched UA and AMI patients. Finally, we explored the changes of metabolites and related metabolic pathways during the course of disease development, providing clues to the physiological and pathological mechanisms involved in disease progression. An overview of the research workflow is shown in Figure 1.

## 2 Methods

### 2.1 Sample collection for LC-MS/MS analysis

The study included 211 participants, 130 ACS patients (103 UA, 27 AMI), and 81 healthy controls. After matched with gender and age, we randomly divided the samples into a discovery group and a validation group (discovery group: validation group = 2:1). The discovery group included 86 ACS patients (69 UA, 17 AMI) and 54HCs. The validation group included 44 ACS patients (34 UA, 10 AMI) and 27 HCs. The clinical characteristics of the patients in the discovery and validation groups are shown in Table 1.

**TABLE 1:** Baseline characteristics of the population.

	HC (n = 81)	UA (n = 103)	AMI (n = 27)	*p*
Age (years)^+^	61.77 ± 14.33	63.64 ± 9.55	63.37 ± 13.04	0.525
Male^&^	47 (50.62)	49 (47.57)	18 (66.67)	0.138
Smoking	28 (34.57)	33 (32.04)	11 (40.74)	0.697
Hypertension^&^	0 (0.00)	49 (47.57)	10 (37.04)	<0.001^ab^
Diabetes mellitus^&^	0 (0.00)	24 (23.30)	11 (40.74)	<0.021^abc^
Hyperlipidemia^&^	0 (0.00)	5 (4.85)	3 (11.11)	0.020^ab^
Laboratory data				
Myo (ng/mL)*	NA	48.86 (28.15–58.80)	116.38 (42.40–100.00)	<0.001^c^
I (TnI) (ng/mL)*	NA	0.10 (0.10–0.10)	2.20 (0.01–0.85)	0.008^c^
CK-MB (ng/mL)*	NA	6.26 (2.00–5.83)	16.77 (2.00–17.70)	0.003^c^
BUN (mmol/L)*	5.51 (4.51–6.30)	5.80 (4.81–7.26)	5.68 (4.38–6.63)	0.153
Scr (μmol/L)*	70.00 (61.84–82.29)	70.80 (59.27–85.25)	79.30 (67.90–91.70)	0.252
Glucose (mmol/L)*	5.42 (5.10–5.79)	5.59 (5.08–6.14)	6.06 (5.15–7.56)	0.017^bc^
AST (U/L)*	24.57 (19.88–29.85)	21.69 (17.24–25.98)	31.33 (20.34–65.68)	<0.001^bc^
ALT (U/L)*	20.67 (14.18–28.62)	18.08 (12.10–27.81)	20.71 (13.18–45.48)	0.396
TG (mmol/L)*	1.42 (1.04–1.88)	1.39 (0.98–2.13)	1.52 (1.07–2.32)	0.209
TC (mmol/L)*	5.35 (4.69–6.05)	4.70 (3.72–5.49)	4.71 (3.81–5.77)	0.001^a^
HDL-C (mmol/L)*	1.41 (1.20–1.60)	1.15 (0.94–1.33)	0.99 (0.87–1.12)	<0.001^abc^
LDL-C (mmol/L)*	3.08 (2.59–3.67)	2.84 (2.14–3.40)	3.02 (2.33–4.14)	0.061

^+^Mean ± SD, * median (IQR),^&^n (%), ^a^ *p* < 0.05 for equality between HC and UA patients. ^b^ *p* < 0.05 for equality between HC and AMI patients. ^c^ *p*< 0.05 for equality between the UA and AMI patients.

All patients in this study were diagnosed with ACS by coronary angiography. The diagnosis criteria for STEMI include: persistent chest pain; ECG ST segment arch upward elevation; and troponin cTnT or cTnI positivity. The clinical diagnostic criteria for UA and NSTEMI are as follows: no ST segment elevation on ECG; elevated CKMB; a negative troponin cTnT or cTcI is UA, and a positive troponin cTnT or cTcI is NSTEMI (Byrne et al., 2024). Patients with atypical chest discomfort also underwent coronary angiography, and patients without stenosis and other major diseases were healthy controls. Patients with malignant tumors, autoimmune diseases, infectious diseases or severe renal insufficiency were excluded.

This study was approved by the Ethics Committee of the China-Japan Union Hospital of Jilin University (approval number:2023072604), and all the subjects provided informed consent before participating in this study. Urine samples were collected after the first urination in the morning after admission. The samples were centrifuged for 10 min at 3,000×g at 4°C within 6 h after collection. The supernatants were separated and stored at −80°C until analysis.

### 2.2 Sample preparation for LC-MS/MS analysis

For urine metabolomics, acetonitrile (200 µL) was added to each urine sample (200 µL), which was then rotated for 30 s and centrifuged at 14,000 × g for 10 min. Then, thesupernatant was vacuum dried, and the vacuum drying mixture was redissolved in 200 µL of 2% acetonitrile. Finally, the urine metabolites were further separated from the macromolecules via an ultracentrifugation filter (Millipore Amcon Ultra (MA)) with a truncated molecular weight of 10 kDa and transferred to an automatic injector.

The quality control (QC) samples were mixed urine samples. All samples from different analysis groups were mixed with 1 µL each as QC samples and are therefore globally representative. The same mass spectrometry method was used for the QC and other urine samples. Throughout the analysis, QC samples are injected once every ten samples to provide data against which the overall method stability and reproducibility can be assessed.

### 2.3 Online HPLC and LC-MS/MS for metabolomics

Ultrahigh performance LC-MS analysis of the urine samples was performed using the Waters ACQUITY Stage liquid chromatography system and an Orbitrap Fusion Lumos Hybrid mass spectrometer (Thermo Fisher Science, Massachusetts, United States). The metabolite separation column used was a Waters HSS C18 (3.0 × 100 mm, 1.7 μm) with gradient elution at a flow rate of 0.5 mL/min. The column temperature was set to 50°C. Mobile phase A was 0.1% formic acid aqueous solution, and mobile phase B was acetonitrile. The mass spectrometer works in positive ion mode, with a scanning speed of 100–1,000 m/z and a resolution of 60k. The automatic gain control (AGC) target was 1 × 10^6^ and the maximum injection time was 100 ms. Then, the QC samples were subjected to UPLC-targeted MS/MS analysis to identify the differential metabolites. The resolution was 15 K, the AGC target was 5 × 10^5^, the maximum information transfer time was 50 ms, and the isolation window width was 3 m/z. Depending on the target for each collisional dissociation (HCD) fragment with higher energy, 20, 40, and 60 are set as the optimal collision energies.

### 2.4 Statistical analysis and data processing

Progenesis QI (Waters, Milford, MA) software is used for raw data normalization and recognition (Zhang et al., 2016). The raw data normalization process includes the calculation of scalar factors using ratiometric data in log space, along with a median and mean absolute deviation outlier filtering approach. Moreover the recognition process includes sample comparison, peak selection, peak grouping, deconvolution and final information output. Massive list data files are exported from QI. The detailed workflow for Progenesis QI raw data normalization, data processing and metabolite identification is given in Supplementary Material S1, S2. Missing value evaluation, logarithm transformation, Pareto scaling and other preprocessing are performed in MetaboAnalyst 6.0 (https://www.metaboanalyst.ca), where variables in the samples with 50% or more missing values are excluded, the missing values within the group are filled with the KNN method, and the missing values between the groups are filled with the minimum value. Principal component analysis (PCA) and orthogonal partial least squares discriminant analysis (OPLS-DA) were performed using SIMCA 14.1 (Umetrics, Sweden) software. To avoid overfitting the model, 200 permutations were used to verify the OPLS-DA model. Non-parametric tests (Wilcoxon RAKSUM test) were used to evaluate the significance of disease-related variables. The Benjamini method was used to estimate the probability of false positives, and explain multiple test comparisons, and the FDR cutoff value was 0.05. The selection criteria for differential metabolites were a p-value <0.05 (nonparameter Wilcoxon rank-sum test) and a fold change≥1.5. The appropriate metabolite group was selected in the training group, the logistic regression algorithm was used to construct the corresponding ROC curve of the metabolite group, and its accuracy was verified in the verification group. Metabolite annotation and pathway Analysis can be performed in the MetaboAnalyst 6.0 platform. The obtained spectra were imported into Progensis QI and significantly different metabolites were further identifiedon the basis of compound ID, adduct, formula, fraction, mass error (Ppm), isotopic similarity, theoretical isotopic distribution, m/z values and MS/MS fragments matched to databases (HMDB, Metlin and mzCloud). A range of scores from 0 to 60 was used to quantify the reliability of each identity. The threshold was set at 35.0. Metabolite pathway analysis and enrichment analysis were performed using the metabolomics visualization web tool MetaboAnalyst 6.0.

## 3 Results

### 3.1 Subjects

A total of 211 samples were analyzed in this study, including 130 patients with ACS (103 UA, 27 AMI) and 81 healthy controls. The 211 samples were matched with gender and age before randomly divided into discovery group (69 UA,17 AMI, 54 HCs) and a validation group (34 UA,10 AMI, 27 HCs). All groups were matched in terms of age and sex, and smoking status was not statistical differences. As shown in Table 1, for some traditional cardiovascular risk factors, such as hypertension, diabetes and hyperlipidemia, UA and AMI were more common than in HCs (*p* < 0.05). The levels of Myo, I (TnI) and CK-MB myocardial injury markers in AMI patients were higher than UA patients (*p* < 0.05). UA patients had higher levels of total cholesterol (TC) and low-density lipoprotein cholesterol (LDL) than HC patients (*p* < 0.05). High density lipoprotein (HDL) levels were lower in patients with AMI than in patients with metastatic disease UA and HCs (*p* < 0.05). Aspartate aminotransferase (AST) levels increased as the disease progressed (*p* < 0.05). These results indicate that with increasing disease severity, the inflammatory response and metabolic system are disrupted.

### 3.2 Quality control

In this study, QC samples are injected once every ten samples. QC correlation can be used to evaluate the reproducibility of instrumental analysis. A total of nine QC samples were injected. The correlation analysis of the QC samples (Supplementary Figure S1) revealed r values (correlation coefficients) close to 1, demonstrating the quality of the QC data and the reproducibility of the instrumental analysis. Therefore, the intergroup differences in the experiment mainly originated from metabolic changes between samples, excluding the influence of other factors. The median of metabolite CV was 0.29.

### 3.3 Differential analysis of ACS metabolomics in urine metabolomics

**FIGURE 2:**
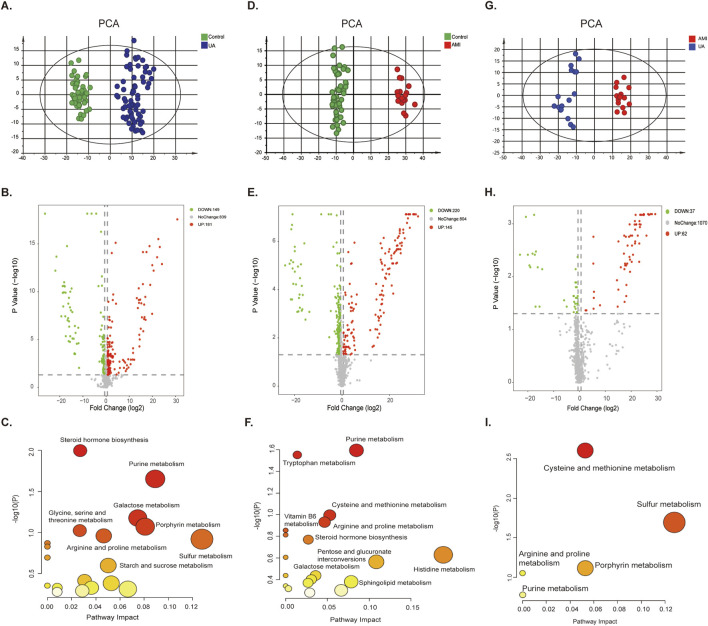
Differential analysis of ACS metabolomics in urine. [**(A–C)** UA vs. HC. **(D–F)** AMI vs. HC. **(G–I)** UA vs. AMI.] **(A, D, G)** PCA score plot for the discovery group. **(B, E, H)** The volcano plots of the identified metabolites. **(C, F, I)** Pathway analysis of differential features.

LC-MS-based analysis revealed that the urine samples yielded 1169 metabolite features (Supplementary Table S1). Multivariate statistical analysis model was used to screen potential biomarkers of UA, AMI and HCs. Principal component analysis (PCA) (Figures 2A, D, G) and orthogonal partial least squares analysis (OPLS-DA) models (Supplementary Figures S2A–C) for both UA and AMI vs. HCs and UA vs. AMI showed a significant clustering trend between the two groups, demonstrating that there are differences among the three groups. The permutation test was used to verify the stability of the regulatory model proposed in this study (Supplementary Figures S2D–F). By adjusting the pvalue <0.05 (nonparameter Wilcoxon rank-sum test) and fold change≥1.5 as the criteria for identifying differential metabolites, a total of 330 (UA vs. HCs), 365 (AMI vs. HCs) and 99 (UA vs. AMI) significantly different metabolites were identified (Figures 2B, E, H; Supplementary Table S1). Pathway analysis of the differential metabolites revealed that the differential metabolites in the UA group were associated mainly with galactose metabolism and steroid hormone biosynthesis (Figure 2C). The differential metabolites in the AMI group were associated mainly with the amino acid metabolism pathway (Figure 2F). The metabolic pathways of cysteine and methionine metabolism and sulfur metabolism changed between UA and AMI (Figure 2I). Both groups presented with disturbances in purine metabolism and porphyrin metabolism pathways.

**FIGURE 3:**
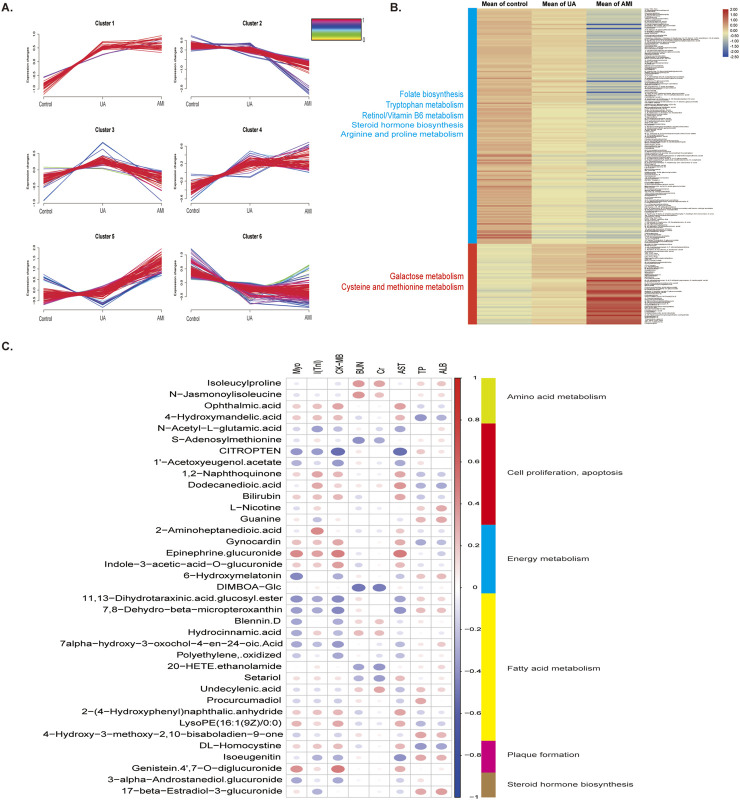
Changes in metabolites during disease progression and correlations between clinical indicators **(A)** Mfuzz correlation analysis of metabolites associated with different degrees of ACS. The color bar represents the Z -score change from 0 to 1. **(B)** Heatmap of differential metabolites in clusters 2,6,4, and 5 in the MFUZZ analysis. **(C)** Correlation analysis between metabolomic profiles and the ACS phenotype.

Figure 3A shows the Mfuzz cluster analysis of 512 differential urinary metabolites in the three groups. Among the six clusters (Supplementary Table S2), most of the metabolites in clusters 2 (94) and 6 (163) decreased with ACS progression and were enriched mainly in retinol metabolism, tryptophan metabolism and amino acid metabolism. Metabolites in clusters 4 (73) and 5 (87) were positively correlated with ACS progression, and were enriched mainly in galactose metabolism, and cysteine and methionine metabolism (Figure 3B). To further explore the relationships between differential metabolites and ACS phenotypes, we performed Pearson correlation analyses of ACS-related clinical indicators such as myoglobin (MyO), troponin I (cTnl), creatine kinase-MB(CK-MB), aspartate aminotransferase (AST), blood urea nitrogen (BUN),and creatinine (Cr) with differential metabolites (Figure 3C; Supplementary Table S3). The results showed that S-adenosylmethionine was positively correlated with troponinI I (cTnl) and negatively correlated with BUN and Cr. Homocysteine has been identified as an independent risk factor for vascular disease, and s -adenosylhomocysteine, a precursor of homocysteine, may also be a sensitive risk indicator, which may be associated with myocardial damage, and therefore positively correlated with I (cTnl) (Kerins et al., 2001; Elshorbagy et al., 2013). Correlation analysis also revealed that CITROPTEN was negatively correlated with MyO, troponin, CK-MB, and AST. CITROPTEN can inhibit the proliferation and migration of vascular smooth muscle cells by binding to receptors on smooth muscle cells, which are associated with arterial remodeling, thus, CITROPTEN may be associated with angiogenesis and myocardial remodeling after myocardial injury (Pham et al., 2024). 4-Hydrocinnamic acid, bilirubin and DL-homocysteine are positively correlated with MyO, troponin, CK-MB, and AST.

### 3.4 Diagnostic biomarkers to identify ACS

**FIGURE 4:**
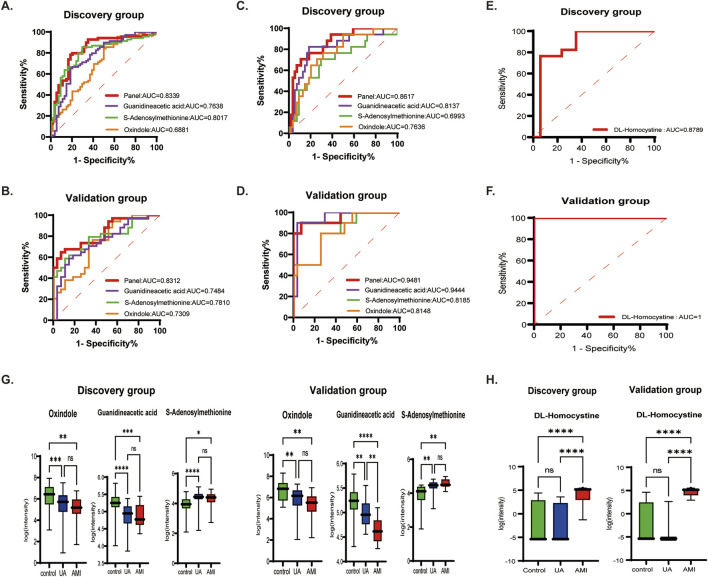
Biomarkers that distinguish ACS from HC, AMI from UA [**(A, B)** UA vs. HC. **(C, D)** AMI vs. HC. **(E, F)** UA vs. AMI.] **(A, C, E)** ROC cure of candidate biomarkers and predictive panel in the discovery group. **(B, D, F)** ROC curves of the candidate biomarkers and the predictive panel in the validation group. **(G, H)** Boxplots showing the expression of the candidate biomarkers for ACS vs. HCs and AMI vs. UA in the panel.

A total of 182 differential metabolites were found in both the UA and AMI groups compared with HCs. Among them, 60 endogenous differential metabolites were identified, and from the pathway analysis and MS/MS analysis, we defined three differential metabolites, guanidineacetic acid, S-adenosylmethionine, oxindole, as potential biomarkers (Supplementary Figure S3). Predictive models were obtained on the basis of exploratory analysis ofreceiver operating characteristic (ROC) curves and logistic regression algorithms. The results revealed that the area under the ROC area of the panel consisting of guanidineacetic acid, S-adenosylmethionine, oxindole was 0.8339 (UA patients vs. HCs) and 0.8617 (AMI patients vs. HCs) (Figures 4A, C; Table 2). The model was then validated with validation sample cohorts (34 UA, 10 AMI and 27 HCs) and area under the curve (AUC) values of 0.8312 and 0.9481 were obtained (Figures 4B, D;Table 2). Box plots show the expression of these three metabolites in the control, UA and AMI groups, with consistent expression in the discovery and validation groups (Figure 4G; Table 2). Moreover, we found that among the differential metabolites, DL-homocystine has a very good capacity for AMI and UA, which was validated in the validation group by exploratory analysis of ROC curves with an area under the curve of 0.8789, which was validated in the validation group with an AUC value of 1 (Figures 4E, F; Table 2). Box plots show that the expression of DL-homocystine in the control, UA and AMI groups was consistent with that in the discovery and validation groups (Figure 4H; Table 2).

**TABLE 2:** Performance of the candidate biomarker panel for ACS.

Exposure	AUC	Sensitivity	Specificity
UA VS HC (Discovery group)			
Panel	0.8339 (0.7591–0.9086)	0.7826 (0.6718–0.8636)	0.8148 (0.6916–0.8962)
Guanidineacetic acid	0.7638 (0.6766–0.8511)	0.6522 (0.5345–0.7538)	0.8148 (0.6916–0.8962)
S-Adenosylmethionine	0.8017 (0.7213–0.8820)	0.8551 (0.7534–0.9193)	0.7037 (0.5717–0.8086)
Oxindole	0.6881 (0.5927–0.7835)	0.8406 (0.7367–0.9086)	0.5000 (0.3711–0.6289)
UA VS HC (Validation group)			
Panel	0.8312 (0.7310–0.9313)	0.6471 (0.4791–0.7851)	0.9259 (0.7663–0.9868)
Guanidineacetic acid	0.7484 (0.6244–0.8723)	0.5884 (0.4222–0.7363)	0.8519 (0.6752–0.9408)
S-Adenosylmethionine	0.7810 (0.6668–0.8953)	0.5882 (0.4222–0.7363)	0.8889 (0.7194–0.9615)
Oxindole	0.7309 (0.6031–0.8588)	0.7647 (0.6000–0.8756)	0.6296 (0.4423–0.7847)
AMI VS HC (Discovery group)			
Panel	0.8617 (0.7656–0.9577)	0.7059 (0.4687–0.8672)	0.8889 (0.7781–0.9481)
Guanidineacetic acid	0.8137 (0.6914–0.9361)	0.8235 (0.5897–0.9381)	0.8148 (0.6916–0.8962)
S-Adenosylmethionine	0.6993 (0.5494–0.8493)	0.7050 (0.4687–0.8672)	0.7037 (0.5717–0.8086)
Oxindole	0.7636 (0.6420–0.8852)	0.7647 (0.5274–0.9044)	0.6852 (0.5526–0.7932)
AMI VS HC (Validation group)			
Panel	0.9481 (0.8603–1.000)	0.9000 (0.5958–0.9949)	0.9259 (0.7663–0.9868)
Guanidineacetic acid	0.9444 (0.8658–1.000)	0.9000 (0.5958–0.9949)	0.9630 (0.8172–0.9981)
S-Adenosylmethionine	0.8185 (0.6672–0.9699)	0.8000 (0.4902–0.9645)	0.7407 (0.5532–0.8683)
Oxindole	0.8148 (0.6644–0.9652)	0.8000 (0.4902–0.9645)	0.7407 (0.5532–0.8683)
UA VS AMI (Discovery group)			
DL-Homocystine	0.8789 (0.7505–1.000)	0.7647 (0.5274–0.9044)	0.9412 (0.7302–0.9970)
UA VS AMI (Validation group)			
DL-Homocystine	1.000 (1.000–1.000)	1.0000 (0.7225–1.0000)	0.9000 (0.5958–0.9949)

Panel*: combination of Guanidineacetic acid, S-Adenosylmethionine, Oxindole.

## 4 Discussion

**FIGURE 5:**
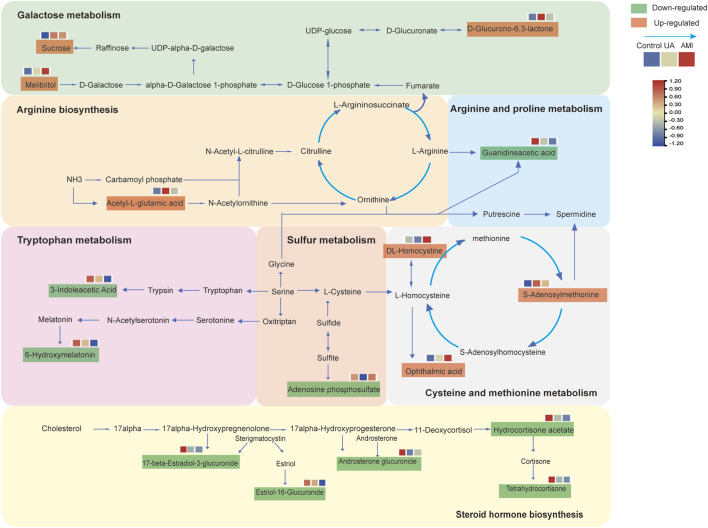
Metabolic pathways of disorders associated with the pathogenesis of ACS.

In this study, we applied the LC-MS technique to perform metabolomic analysis of urine from patients with ACS and healthy controls. Through the comparison of UA, AMI and health, we found that ACSs were associated mainly with ACSs were associated mainly with arginine biosynthesis, cysteine and methionine metabolism, galactose metabolism, sulfur metabolism, steroid hormone biosynthesis and other pathway changes (Figure 5). A panel of guanidineacetic acid, S-adenosylmethionine, oxindole could distinguish ACS patients from healthy controls. Moreover, we also found that DL-homocystine has the ability to distinguish between UA and AMI. With respect to MFUZZ, we found that the metabolites whose levels increased with disease severity were involved mainly in cysteine and methionine metabolism, and galactose metabolic pathways. Metabolites whose abundance decreases with disease severity are associated mainly with tryptophan metabolism.

### 4.1 Differences in urine metabolomics in patients with ACS

Tryptophan metabolism, cysteine and methionine metabolism, arginine biosynthesis, steroid hormone biosynthesis, and galactose metabolism are associated with the development of ACS. These metabolic pathways are closely linked to the development of the disease (Surendran et al., 2021).

Fatty acid and glucose metabolism is known to be required for ATP to reach the heart. In addition, ATP can also come from galactose, a small number of ketone bodies and amino acids (Doenst et al., 2013). Previous studies have shown found that metabolomic analysis of different biological samples of AMI revealed changes in fatty acid and galactose metabolism (Wu et al., 2020a), which was consistent with our results. Studies have also shown that under normal circumstances, galactose is converted into glucose to participate in human metabolism, but when galactose is excessive, it metabolizes ROS, thereby inducing oxidative stress and inflammation, and regulating cell proliferation and apoptosis (Qi and Tester, 2019; Shi et al., 2024).

Sulfur REDOX signaling has long been considered a key mechanism in the development of heart disease (Go and Jones, 2013). Cardiomyocytes have a good antioxidant system, which is mostly based on cysteine (Cys) (Nishimura et al., 2022). H_2_S can be used as an endogenous neuromodulator and vascular relaxant (Abe and Kimura, 1996; Li et al., 2011). Moreover, the discovery of supersulfide and its biochemical properties have raised the possibility that it has cardioprotective effects as a molecular entity (Nishimura et al., 2024). This corresponds to the downregulation of sulphur metabolism when myocardial hypoxia occurred in our study. Myocardial damage occurs when the blood supply to the heart is briefly blocked by ischemia and then restored (Elrod et al., 2007). Rapid oxygen reentry induces an increase in mitochondrial ROS, leading to cardiac cell death and tissue damage (Osipov et al., 2009). Experimental studies have shown that sulfur metabolites can improve myocardial hypertrophy by reducing oxidative stress depending on the PI3K-Akt signaling pathway (Li et al., 2018; Luan et al., 2012; Kondo et al., 2013). These findings indicate that sulfur metabolites have the most protective effect on the myocardium. In our study, the decreased expression of sulfur metabolites during UA exposure may indicate the occurrence of oxidative stress and eventual damage the myocardium.

### 4.2 Differences in urine metabolomics between UA and AMI patients

Through urine metabolomics analysis of ACS, it was found that the expression of metabolites in the steroid hormone biosynthesis pathway gradually decreased with increasing disease severity. Previous studies have also shown that low levels of estrogen, or testosterone, can increase the risk of atherosclerosis and coronary heart disease. This finding is consistent with our results. Moreover, Hu et al. (2011), Akishita et al. (2010), and Malkin et al. (2003). The testosterone levels in the UA and AMI groups were significantly lower than those in the SAP group. The level of testosterone in the AMI group was significantly lower than that in the UA group. These experimental results further confirmed that with increasing disease severity, the expression of steroid hormone-related metabolites decreases. Decreased testosterone levels are associated with increased systemic vascular resistance, decreased heart rate variability, and decreased baroreflex sensitivity (Chen et al., 2014). It can also induce coronary artery relaxation (Chen et al., 2014). In addition, researchers have confirmed that hormone levels are related to the severity of heart ischemia and hypoxia (Bell et al., 2011). Estrogen is inversely associated with heart attack size, and estrogen use significantly reduces heart attack size in men (Ostadal and Ostadal, 2014).

Our results also revealed that the tryptophan metabolism pathway changed when ACS occurred. In 2019, jia et al. used UPLC-QTOF-MS to conduct metabolomics analysis of rat serum after MI and reported that phospholipid metabolism, sphingolipid metabolism and linoleic acid metabolism increased, and that tryptophan metabolism decreased, which was the same as our results (Jia et al., 2019). Tryptophan metabolites, as important regulators of immune and inflammatory responses, have been shown to play a key role in cardiovascular disease (Nitz et al., 2019). Studies have also shown that tryptophan metabolites downstream of kynurenine are associated with an increased risk of AMI in patients with stable angina pectoris (Pedersen et al., 2015). Moreover, the infarct size of rats treated with the tryptophan metabolites kynurenine and kynurenicuric acid was significantly smaller (Bakhta et al., 2020). These results indicated that tryptophan metabolites were negatively correlated with myocardial infarction size, which was consistent with the finding that the expression of tryptophan metabolites decreased gradually with the development of the disease.

### 4.3 Potential biomarkers for the diagnosis of ACS

In our study, a panel of three urinary metabolites showed good performance in distinguishing patients with ACS from controls. Guanidineacetic acid can be methylated by guanidinoacetate N-methyltransferase (GAMT), which uses S-adenosylmethionine as a methyl donor to produce creatine (Nouioua et al., 2013). Schneider et al. (2008) reported that GAMT is an essential enzyme for creatine biosynthesis and that mice lacking this enzyme cannot synthesize creatine. Creatine plays an important role in buffering and transferring high-energy phosphate bonds in the heart, and creatine deficiency can impair heart function. Guanidinoacetic acid is the raw material of creatine synthesis, so it is speculated that a reduction in guanidinoacetic acid leads to a reduction in creatine, which is harmful to the myocardium. Lorenzo et al. reported that the metabolic pathways of arginine and proline are altered in patients with atherosclerosis and ACS, and can be used as new monitoring tools (Martin-Lorenzo et al., 2015). Loss of nitric oxide is one of the mechanisms of endothelial dysfunction, and providing more substrates for endothalium-specific nitric oxide synthase (eNOS) is the mechanism by which the vascular endothelium increases nitric oxide synthesis (Nicholls et al., 2007). However, arginine is an important substrate of this enzyme, and the level of guanidineacetic acid gradually decreases as acute coronary syndrome exacerbates, resulting in changes in arginine metabolism. A reduction in arginine and its metabolites leads to the loss of nitric oxide, resulting in injury to endothelial function, and leading to the aggravation of ACS disease (Schulman et al., 2006; Bekpinar et al., 2011; Molek et al., 2021).

In addition, S-adenosylmethionine and DL-homocystine can also distinguish ACS and its subtypes. Previous studies have shown that homocysteine is a risk factor for atherosclerotic thrombosis (Becker et al., 2003), a cardiovascular disease risk factor (Zawada et al., 2014). Homocysteine, S-adenosylmethionine, S-adenosylhomocysteine (SAH), DL-homocystine, and methionine are involved in the cysteine and methionine metabolic pathways. Homocysteine is derived from methionine metabolism and SAM dependent transmethylation (Hartz and Schalinske, 2006). Nam et al. (2017) found that the concentration of SAM and the SAM/SAH ratio gradually decreased in a time-dependent manner in the metabolomic analysis of mouse hearts after MI, which was similar to the increase in SAM expression during UA and the decrease in DL-homocystine during AMI. The expression of SAM decreased, whereas the expression of DL-homocystine increased. Experiments have shown that after myocardial infarction, the levels of the SAM dependency methyltransferases Coq3 and Coq5 gradually decrease. Coq3 and Coq5 participate in the COQ biosynthetic pathway (Loehrer et al., 2001). Downregulation of COQ in cardiac tissue after myocardial ischemia can affect aerobic cell respiration and lead to insufficient ATP production in cardiomyocytes (Langsjoen et al., 1994). Puddu (2018) found that homocysteine (Hcy) associated acute myocardial infarction risk may reflect lipid metabolism disturbances. Moreover, when homocysteine levels are elevated, there may be a risk of impaired endothelial function, which increases the possibility of thrombosis, and oxidative stress and inflammatory stimulation can lead to coronary artery calcification, which leads to the occurrence of acute coronary syndrome (Martí-Carvajal et al., 2015). Elevated homocysteine levels are be closely related to inflammatory responses. Homocysteine can exacerbate the inflammatory process by activating Th1 and Th17 cells and promoting the release of pro-inflammatory cytokines (Sitdikova and Hermann, 2023). In ACS patients, homocysteine levels are positively correlated with a variety of inflammatory markers, such as white blood cell count, neutrophil count, C-reactive protein (CRP), monocyte count, and interleukin-6. This inflammatory response not only aggravates the severity of coronary artery disease, but also may cause plaque instability and increase the risk of thrombosis (Jin et al., 2025). Homocysteine can promote the generation of ROS, which can cause cell dysfunction by damaging cell membranes, proteins and DNA (Liu et al., 2024). In addition, elevated levels of homocysteine can induce endoplasmic reticulum stress, which then activates the apoptotic pathway, leading to damage of to vascular endothelial cells and smooth muscle cells (Calim et al., 2020). Most importantly, homocysteine is able to activate the inflammatory response and further aggravate oxidative stress. This process plays a key role in the progression of atherosclerosis (Pushpakumar et al., 2014). DL-homocystine is the oxidation product of homocysteine. From the above discussion, we know that oxidative stress is a key factor in the pathophysiological process of UA and AMI. When the level of Hcy gradually increases during disease progression, the level of DL-homocystine, the oxidative product of Hcy, may significantly increase under oxidative stress. As shown in Figure 5,we mapped metabolic pathways on the basis of the altered metabolic pathways identified in the present study.

Through our metabolomic study of urine in from ACS, we found that the metabolite oleic Acid is altered during the course of the disease. Yin et al. (2017) and Gundogdu et al. performed metabolomic analysis of patients with ACS using plasma and serum samples, and also detected changes in oleic Acid. Zhu et al. (2018) used the metabolomics method revealed find that C16-sphingosine in plasma was altered in AMI patients, which was the same as our experimental results. In addition, metabolites such as 5′-adenylic acid (Cebo et al., 2022), decanoylcarnitine (Zhou et al., 2024), and 5-hydroxyindoleacid (Wu et al., 2020b) have been found to be altered in blood metabolomics studies of ACS. These findings suggest that metabolomic studies of urine can reveal changes in metabolites. And Urine has the advantages of being noninvasive and easy to obtain and store. However, it is undeniable that combining urine and blood results will cover a more comprehensive range of metabolite, significantly improving the depth and breadth of metabolic research. There are also similar problems in the technical aspects of metabolomics. In this study, UPLC-MS technology was used for metabolomics analysis. There are a variety of techniques for metabolomics, including NMR, and GC-MS. Compared with GC-MS and LC-MS, NMR is a pioneer platform for metabolomics, with advantages such as high repeatability and suitability for quantitative analysis with fewer sample requirements (Sengupta and Weljie, 2019), while LC-MS can detect metabolites without the limitations of volatility and thermal instability, and sample preparation is simpler than GC-MS, does not require derivatization, and is more sensitive (Li et al., 2019). Khan et al. (2020) also revealed the changes in L-homocysteine levels via the application of HRM technology for metabolomic analysis of the blood of AMI patients. Hypoxanthine, urea, etc. were found to have altered metabolites in our study, which was also found in previous experiments based on HRM (Deng et al., 2018; Ali et al., 2016). Therefore, the metabolites found by different techniques were partially the same, but the multi-technique association made the results more abundant.

There are several limitations to this study. First, with respect to the effects of bacteria on urine metabolomics, we have chose to midstream urine to reduce microbial contamination of the urethral opening during urine collection. At the same time, the collected urine samples were centrifuged (3,000×g, 10 min) to remove the microorganisms and cell debris in the urine, and the supernatant urine was taken for short-term storage at - 80°C. The above methods can effectively reduce the influence of microorganisms on urine metabolomics and improve the accuracy and reliability of the study results. However, some potential factors produced by microorganisms can affect metabolome analysis. Metabolites produced by bacteria directly alter the composition of urine metabolites, and bacteria may consume certain metabolites in the urine, resulting in changes in the concentrations of these metabolites in the urine (Zheng et al., 2024; Yang et al., 2023; Queremel Milani and Jialal, 2025). The intestinal flora affects the body’s systemic metabolism through metabolites (such as short-chain fatty acids and indole compounds), and thus affects urine metabolomics (Dong et al., 2023). In future studies, 16S rRNA sequencing could be used to determine whether bacterial infections are present, and the effects of the gut flora on metabolites throughout the body should be further studied. In addition, our study sample came from a center primarily used for non-targeted LC-MS/MS analysis, and the differential metabolites found provide preliminary results for potential candidate biomarkers. Therefore, multi-center samples can be collected in future experiments, and targeted metabolomics can be used to further verify experimental results while non-targeted high-throughput screening of differential metabolites. Furthermore, in our study, only the urine of patients with ACS was analyzed by LC-MS/MS. In future experiments, we will apply a variety of metabolomics technologies to analyze biological fluids together to further strengthen the research results. Finally, the functions and mechanisms of the differential metabolites identified in these experiments are still unclear and can be explored through biochemical research methods such as *in vivo* and *in vitro*.

## 5 Conclusion

In this study, we used LC-MS to define the metabolome of urine samples from ACS patients and healthy controls. The results show that the urinary metabolome can distinguish patients with ACS from healthy controls and reveal changes in metabolic expression levels over the course of the disease. In addition, the panel of metabolites identified can be used as potential diagnostic biomarkers to distinguish ACS patients from healthy controls. This study may provide a new perspective for the differential diagnosis and monitoring of ACS.

## Data Availability

Data that support the findings of this study have been deposited in integrated proteome resources (https://www.iprox.cn/) with Project ID IPX0010403000, PXD058501.

## Supplementary material

SUPPLEMENTARY TABLE S1
Differential metabolites in each cohort.

SUPPLEMENTARY TABLE S2
Results of MFUZZ.

SUPPLEMENTARY TABLE S3
Pearson correlations between metabolites and the ACS phenotype.

## Abbreviations

ACS, Acute coronary syndrome; LC-MS, liquid chromatography-mass spectrometry; UA, unstable angina; AMI, acute myocardial infarction; STEMI, ST-segment elevation myocardial infarction; NSTEMI, non-ST-segment elevation myocardial infarction; GC-MC, gas chromatography-mass spectrometry; UPLC/MS, Ultra-performance liquid chromatography/mass spectrometry; QC, Quality control; PCA, principal component analysis; OPLS-DA, orthogonal Partial least squares discriminant analysis; TC, total cholesterol; LDL, low-density lipoprotein cholesterol; HDL, High density lipoprotein; AST, Aspartate aminotransferase; MyO, Myoglobin; CK-MB, creatine kinase-MB; Cr, Creatinine; BUN, Blood Urea Nitrogen; AS, atherosclerosis; Cys, cysteine; GAMT, guanidinoacetate N-methyltransferase; SAH, S-adenosylhomocysteine; AUC, Area Under Curve; ROC, Receiver Operating Characteristic Curve.
