# Supplementary material for: Urinary metabolomics analysis based on LC-MS for the diagnosis and monitoring of acute coronary syndrome
Source: Front Mol Biosci. 2025 Apr 9;12:1547476. doi: 10.3389/fmolb.2025.1547476 (PMC12014464; doi:10.3389/fmolb.2025.1547476)
Supplement: Supplementary file 4 [file DataSheet1.docx]

Supplementary Material

Urinary metabolomics analysis based on LC-MS for diagnosis and monitoring of Acute coronary syndrome

Jiaqi Liu^1+^, Aiwei Wang^2^, Feng Qi^2^, Xiaoyan Liu^2^, Zhengguang Guo^2^,Haidan Sun^2^, Mindi Zhao^3^, Tingmiao Li^1^,Fei Xue^1^, Hai Wang^1*^,Wei Sun^2*^, Chengyan He^1*^

1Department of Laboratory Medicine, China-Japan Union Hospital of Jilin University,

Changchun, China

2Institute of Basic Medical Sciences, Chinese Academy of Medical Sciences, School of Basic Medicine, Peking Union Medical College, Beijing, China

3 Department of Laboratory Medicine, Beijing Hospital, National Center of Gerontology; Institute of Geriatric Medicine, Chinese Academy of Medical Sciences, Beijing, China

*Correspondence:

Hai Wang, wanghai@jlu.edu.cn

Wei Sun, sunwei@ibms.pumc.edu.cn

Chengyan He, cyhe@jlu.edu.cn

1. Process - Normalise to all compounds using Progenesis QI
2. Normalisation reference (the 'target')

One run is automatically selected as the normalisation reference.

1. Log10 ratio calculation

Because of the accurate alignment and aggregate co-detection, every run has a reading for all compound ions. Hence, for every run, a ratio can be taken for the value of the compound ion abundance in that run to the value in the normalisation reference: ‘*Ri,x=Abi,x/Abi,NR’* Where ‘*Ri,x’* is the ratio of the abundance of the compound ion *‘i*’ in run ‘*x’* to that of compound ion *‘i’* in the normalisation reference ‘*NR’.* However, such ratiometric data follow a skewed distribution (a 2-fold increase giving 2, a 2-fold decrease giving 0.5; 3-fold giving 3 and 0.33, etc.). To obtain a distribution treating both directions equally, log transformation is applied, which yields a normal distribution. Progenesis QI carries this transformation out (base 10) on all ratio data within each run, and for all runs, to generate a series of normal distributions. At this stage, these are offset – that is, because of scalar differences in signal, the ratios will not centre on 1 (and the log ratios not on zero), as would be the case if there was no global shift in the signal. This is the shift that must be addressed.

1. Scalar estimation in log space

The next step is to centre the log10 ratio distributions onto that of the normalisation reference in each case. This is achieved by simply adding or subtracting the value required to shift the sample distribution over the normalisation reference one. This additive or subtractive shift in log space, is, of course, a multiplicative scalar in the sample abundance space. There is a second improvement over traditional methods applied in this step. The median and also median absolute deviation are used as an approximation of the variance of the ratio distribution; this allows the filtering out of outlying ratio values so that they do not perturb the results. This process is carried out iteratively, to robustly remove the influence of outliers.

1. Scalar application

Once the scalar has been derived in log space and then returned to an ‘abundance-space ratio’, it can be applied to all values in the sample run being normalised, and this completes the process.

2. Data processing using Progenesis QI

The detailed workflow for data processing facilitated by Progenesis QI is involved “create a new experiment”, “import data”, “review alignment”, “experiment design setup”, “peak picking”, “reviewed convolution”, and “identify compounds” in sequence. In general, the whole process ran automatically using optimized parameter settings. (1) In the stage of create a new experiment, adduct ion was carefully selected as it would influence the number of characterized compounds and also the identification accuracy. Based on the ionization behaviors of reference standards, the adduct ion forms, comprising [M + H]+, [M + Na]+, [M + K], [M + NH4]+, [2M + H]+, [2M + Na]+,[2M + NH4]+, [M + H – H2O]+ and [M + H – 2H2O]+ , were selected. (2) The MS data acquired by LC-MS for all the URINE samples were imported into the Progenesis QI software, generating a 2D ion intensity map with the retention time and m/z information as the ordinate and abscissa, respectively. (3) Peak alignment was carried out in automatic manner taking a QC run as the reference, the score values for all the samples were greater than 90 %. (4) For peak picking, the thresholds of chromatographic peak absolute intensity, and retention time limits can be set to achieve the maximum real ion signals with noise excluded. In the present study, absolute intensity and retention time limit were set at 1000 and default. (5) Further compound identification was performed by searching the HMDB database (2018 version) and Metabolite Link (METLIN) and the spectrogram library established by our laboratory using standards. The MS1 mass tolerance was set as 10 ppm and the MS/MS mass tolerance was set as 20 ppm.
