# Supplementary material for: Urinary metabolomics analysis based on LC-MS for the diagnosis and monitoring of acute coronary syndrome
Source: Front Mol Biosci. 2025 Apr 9;12:1547476. doi: 10.3389/fmolb.2025.1547476 (PMC12014464; doi:10.3389/fmolb.2025.1547476)

Fig. S1 Assessment of QC samples. Plot on the QC correlation.

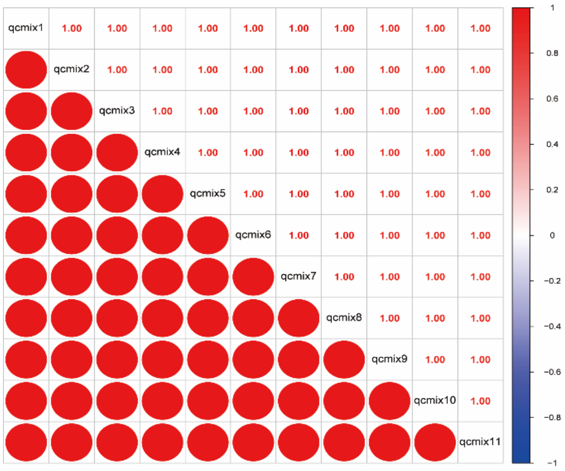

Fig. S2 Differential analysis of ACS metabolomics in urine metabolomics. [(A,D)UA vs HC. (B,E)AMI vs HC. (C,F)UA vs AMI.] (A,B,C) Score plot of OPLS-DA model in the discovery group.（D,E,F） Two-hundred permutation test for the OPLS-DA model in the discovery group.

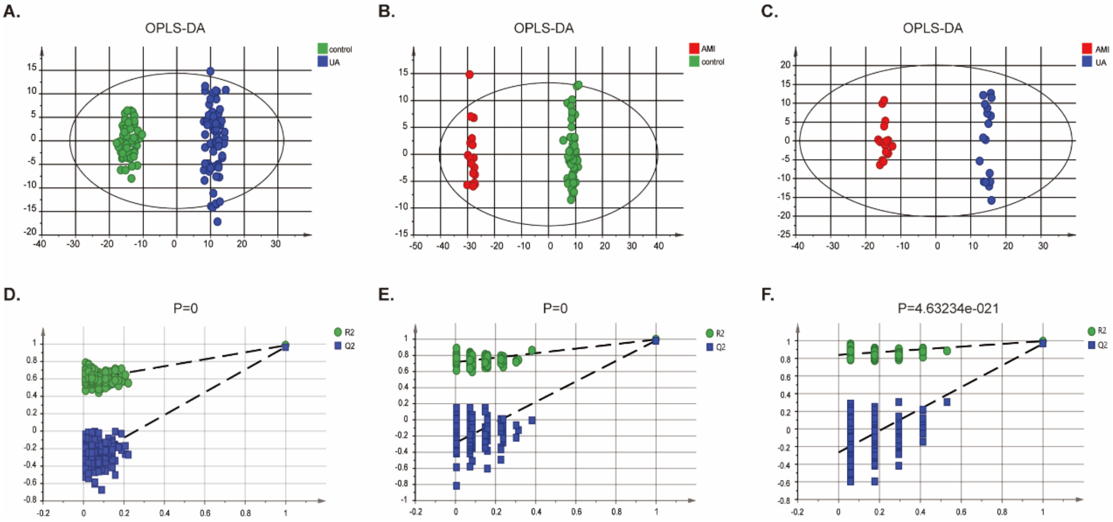

Fig. S3 Flow chart of candidate metabolite selection

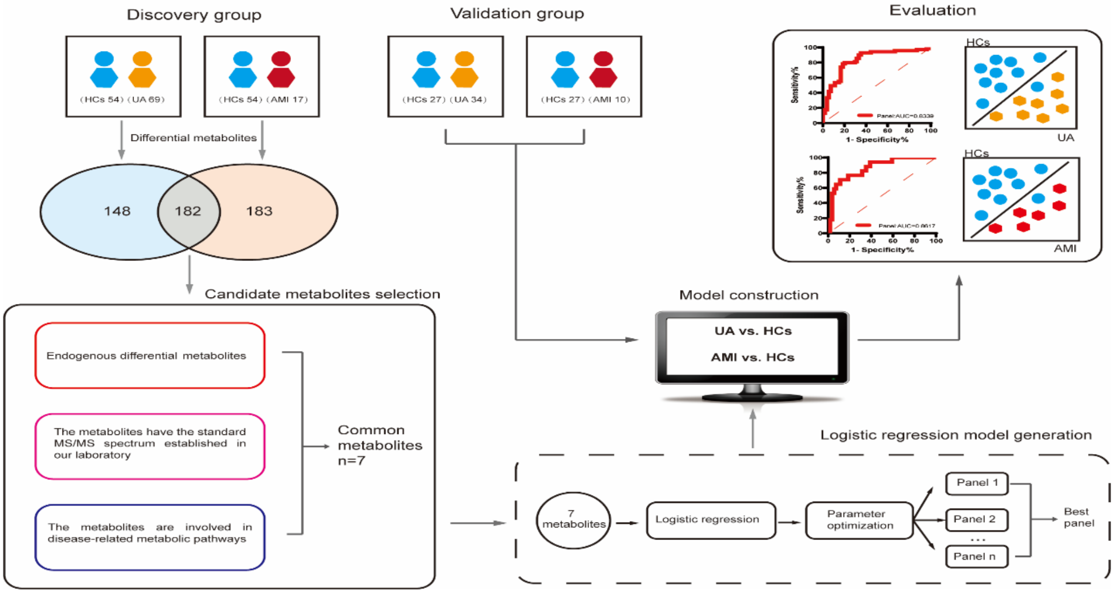

Supplement: Supplementary file 5 [file Image1.pdf]
